# Ultrasound-stimulated microbubbles enhanced vascular disruption in fractionated radiotherapy-treated tumours via ASMase activation

**DOI:** 10.1242/dmm.049531

**Published:** 2023-06-06

**Authors:** Kai Xuan Leong, Wenyi Yang, Deepa Sharma, Stanley Liu, Gregory J. Czarnota

**Affiliations:** ^1^ Physical Sciences, Sunnybrook Research Institute, Toronto, Ontario M4N 3M5, Canada; ^2^ Biological Sciences, Sunnybrook Research Institute, Toronto, Ontario M4N 3M5, Canada; ^3^ Department of Radiation Oncology, Sunnybrook Health Sciences Centre, Toronto, Ontario M4N 3M5, Canada; ^4^ Department of Medical Biophysics, University of Toronto, Toronto, Ontario M5G 1L7, Canada; ^5^ Department of Radiation Oncology, University of Toronto, Toronto, Ontario M5T 1P5, Canada

**Keywords:** Ultrasound stimulated microbubble therapy, PC3 xenograft, Focused ultrasound, Fractionated radiation therapy

## Abstract

Recent studies have indicated that radiotherapy affects tumour vasculature as well as tumour cells. The use of ultrasound-stimulated microbubbles (USMB) can potentially enhance the effects of radiotherapy through the activation of the acid sphingomyelinase [ASMase or sphingomyelin phosphodiesterase 1 (*SMPD1*)]-ceramide pathway. ASMase knockout (ASMase^−/−^) and wild-type (WT) mice bearing fibrosarcoma (MCA/129 tumour line) were treated with 10 Gy or 20 Gy in five fractions alongside or independently of USMB treatments. The results indicated that tumour responses to fractionated radiotherapy (fXRT) were enhanced when fXRT was coupled with USMB as part of the treatment regimen. Sphingosine-1-phosphate (S1P)-treated mice and ASMase^−/−^ mice demonstrated radioresistance against fXRT alone, whereas only ASMase^−/−^ mice showed radioresistance against fXRT treatment alone and when combined with USMB. Results indicated that in WT and S1P-treated cohorts, the use of USMB with fXRT enhanced the tumour response compared to use of USMB or fXRT alone. Although in WT and S1P-treated cohorts, there was enhanced vascular disruption, ASMase^−/−^ cohorts demonstrated no significant vascular disruption, indicating the importance of ASMase in facilitating vascular changes in response to fXRT and USMB treatment.

## Teaser

**Summary:** A novel technique using ultrasound-stimulated microbubbles coupled with fractionated radiation enhanced vascular disruption in the tumour microenvironment via an ASMase-dependent mechanism.

## INTRODUCTION

Ionizing radiation has been used in the clinic to treat a multitude of diseases and most prominently for cancer treatment (Nakamura, 2019). Moderate (0.1-2 Gy) to high (>5-8 Gy) single doses of radiation can cause damage on a cellular level by increasing intracellular oxidative stress and DNA damage. Radiation can inflict DNA damage in many ways, including DNA backbone breakage, DNA base damage, cross-linking and polymerization (Baskar et al., 2012; Helm and Rudel, 2020). DNA double-strand breaks trigger a highly regulated DNA damage repair and response pathway (DDR), which attempts to correct the damaged DNA. As such, the cell undergoes cell cycle arrest to attempt to repair the damage. However, if the damage inflicted is too extensive and DDR is unable to repair the DNA, apoptosis signalling can occur to mark the cell for programmed death. As a result, apoptosis pathways are activated, leading to acute levels of cell death and irreparable damage to tissue and organ structures (Baskar et al., 2012; Helm and Rudel, 2020; Santivasi and Xia, 2014; Streffer, 2015).

Taking advantage of this process, radiation treatments (XRT) are often coupled with other cancer treatments, such as chemotherapy or surgery as an adjuvant or neo-adjuvant treatment. Although large curative doses of radiation (>20 Gy) are extremely effective at eliminating cancer cells, delivery of high doses of radiation comes with risks as surrounding tissue and organ damage can be inflicted through the course of the treatment. Thus, current clinical practices deliver cytotoxic radiation doses (>40 Gy) over the course of several weeks in smaller fractions (1.8-3.0 Gy) to reduce the toxic effects on healthy tissue while simultaneously invoking a curative effect against tumour cells (Baskar et al., 2012). Even with fractionated XRT (fXRT), there come risks of side effects as accumulated damage can lead to adverse side effects. As such, current research is looking for more effective ways of delivering cytotoxic doses of radiation while reducing off target effects.

The conventional theory of radiation-induced cell death establishes the action of ionizing radiation on tumour cells as the primary mechanisms that result in overall tumour control (Baskar et al., 2012; Toulany, 2019; Castle and Kirsch, 2019). Studies conducted by Moding and colleagues demonstrated this by using a dual-recombinase ataxia telangiectasia mutated (ATM)-knockout mouse model to show the effects of single-dose radiotherapy and fXRT in a primary sarcoma model. The results showed that although ATM deletion enhanced endothelial cell death and was capable of delaying tumour regrowth, primary sarcomas exhibited no differences in local tumour control between different genotypic groups (Moding et al., 2014, 2015). This effect is further mediated with the activation of the immune system to produce an anti-tumourigenic effect. XRT-induced stress can lead to the release of signalling components that initiate the mobilization of immune cells, leading to immunogenic cell death of tumour cells (Bezu et al., 2018; Zhu et al., 2021). However, other studies have suggested that tumour cell death is not the primary driving mechanism of radiation-induced cell death, but instead is initiated through the destruction of the tumour vasculature leading to anoxic conditions (Czarnota, 2015; Garcia-Barros et al., 2003; Paris et al., 2001; Corre et al., 2013; Baselet et al., 2019). Indeed, endothelial cells have a greater level of radiosensitivity (to as low as 2 Gy), often resulting in subsequent organ or tissue damage several hours following radiation exposure (Garcia-Barros et al., 2003; Paris et al., 2001).

A study by Garcia-Barros et al. (2003) demonstrated that fibrosarcoma and melanoma tumours showed that increased levels of tumour vasculature disruption caused by a single high-dose XRT (>15 Gy) led to reduced tumour growth and increased levels of cellular apoptosis (Garcia-Barros et al., 2003; Corre et al., 2013). However, at lower doses of radiation (<8 Gy), the lysosomal enzyme acid sphingomyelinase (ASMase, encoded by *SMPD1*) is not released, leading to insignificant vascular response until total cumulative doses go beyond the minimum threshold (Zhu et al., 2015; Fuks and Kolesnick, 2005). Thus, endothelial cell apoptosis is highly modulated by the activation of ASMase, which converts cell-membrane sphingomyelin into ceramide, a crucial secondary messenger for initiating apoptosis (Paris et al., 2001; Corre et al., 2013; Baselet et al., 2019; El-Kaffas et al., 2018). In the study conducted by El-Kaffas et al. (2018), mice with an ASMase knockout (ASMase^−/−^) genotype were shown to exhibit radioresistant phenotypes in which endothelial vasculature was preserved, despite cytotoxic doses of radiation to which these tumours were exposed. Additionally, mice treated with sphingosine-1-phosphate (S1P), a ceramide inhibitor, demonstrated similar outcomes (El-Kaffas et al., 2018). This implicates the importance of ASMase-ceramide activation to facilitate tumour endothelial cell death.

S1P is a sphingolipid that plays an important role in a multitude of functions, including proliferation and the regulation of oxidative stress, and has been associated with cancer progression (Pyne et al., 2018; Pyne and Pyne, 2020). A cellular balance is struck between maintaining ceramide and S1P levels. Excess ceramide can be reduced through the catabolism of ceramide into sphingosine and subsequently phosphorylated by sphingosine kinases to generate S1P or sphingosine-2-phosphate (Pyne et al., 2018; Pyne and Pyne, 2020; Zhao et al., 2020). Given the relationship that S1P has with ceramide in a carefully balanced metabolic process, exogenous S1P can be administered to overwhelm cell signalling to promote cell survival and directly compete against the pro-apoptotic effect that ceramide conveys (Zhao et al., 2020).

Radiation sensitizers or enhancers are sought to make tumours more susceptible to radiation therapy. Studies have shown that ultrasound-stimulated microbubbles (USMB), when used in combination with XRT, can augment the effects of a single high dose of radiation (El-Kaffas et al., 2018). Microbubbles are small gas bubbles (1.0-3.0 µm) often coated in a lipid or protein shell. Gas cores are composed of either perfluorocarbon or sulphur hexafluoride, as they are inert gases with low solubility in the bloodstream (Stride and Coussions, 2010; Chong et al., 2018; Chowdhury et al., 2020). These gases do not cause any adverse effects to the body and are rapidly exhaled within several minutes of dissolution, with the shell being rapidly processed in the liver (Chong et al., 2018). Currently microbubbles are one of a handful of radiological ultrasound contrast agents that have been approved by the US Food and Drug Administration (FDA) to be used clinically to enhance image quality. It is often used in renal or cardiac imaging to visualize heart chambers and vascular networks (Chong et al., 2018). When placed under an ultrasound field, USMBs respond to the acoustic stimulation by compressing and rarefacting. At low-intensity and resonant frequencies, microbubble diameter can increase and decrease in a stable, cyclic pattern, a process known as stable cavitation (Stride and Coussions, 2010). Higher-intensity stimulation causes the bubbles to expand and contract sporadically, no longer oscillating in a stable manner, which can lead to bubble destruction. This is known as inertial cavitation (Stride and Coussions, 2010). Bursting microbubbles using ultrasound has been known to induce damage to endothelial cells lining blood vessels by generating pores in the cell membrane (Chowdhury et al., 2020). Recently, studies have shown that microbubbles combined with ultrasound have multiple potential therapeutic uses, including acting as drug delivery vectors (Hernot and Klibanov, 2008; Al-Jawadi and Thakur, 2020), opening of the blood brain barrier (Lipsman et al., 2018; Carpentier et al., 2016), facilitating the treatment of thrombosis (Alexandrov., 2006; Wang et al., 2016) and as radioenhancers when combined with radiation treatment (Czarnota, 2015; El-Kaffas et al., 2018). The added injury induced by USMB perforation can lead to the activation of ASMase at lower radiation doses at which ASMase is not normally activated, and subsequently initiate endothelial cell apoptosis (Ferranti et al., 2020). These changes to the tumour vasculature can be detected non-invasively through the use of power Doppler (PD) ultrasound.

PD is a non-invasive imaging modality that is used to measure blood flow based on the reflection of ultrasound waves off moving red blood cells and utilizes the principles of the Doppler effect to measure velocity (Satomura, 1957). Current clinical applications of PD are mainly to characterize blood flow in blood vessels to detect ischemia, changes in tissue perfusion and irregular blood flow in tumours (Martinoli et al., 1998; Clautice-Engle and Jeffrey, 1997). Within tumours, the vasculature is often chaotic, disorganized and has non-uniform architectures. In cancer, PD has shown promise in being an effective tool in characterizing tumour vasculature. In solid breast lesions, benign tumours reveal vascular signals of major blood vessels and often show little signal beyond. Malignant tumours, however, have a greater amount of branching and chaotic structure. Signals picked up by PD are displayed as a heat or rainbow map to indicate blood flow intensity and, in the case of a rainbow map, directionality. This, combined with grayscale imaging, can assist in characterizing malignant versus benign lesions in breast cancers and potentially act as a quick and efficient way of monitoring vascular changes in tumours (Ibrahim et al., 2016).

In this study, we investigated the involvement of the ASMase-ceramide pathway in facilitating tumour vascular disruption following USMB and fXRT in C57BL/6 (black 6) mice. Experiments were conducted with wild-type (WT) and ASMase^−/−^ mice implanted with fibrosarcoma xenografts. A group of WT mice receiving the ASMase-ceramide pathway inhibitor S1P was also included. Furthermore, this study aimed to identify the mechanism by which this combined treatment acts in the tumour microenvironment to facilitate the enhanced cell death and vascular damage. Here, we used histological analysis to examine tumour cell death and vascular disruption 72 h after treatment. In addition, PD analysis was used to complement the histology findings by determining the perfusion changes within tumours pre- and post treatment.

## RESULTS

### USMB and fXRT effects on tumour vascularity in WT and S1P-treated mice

**Fig. 1.**
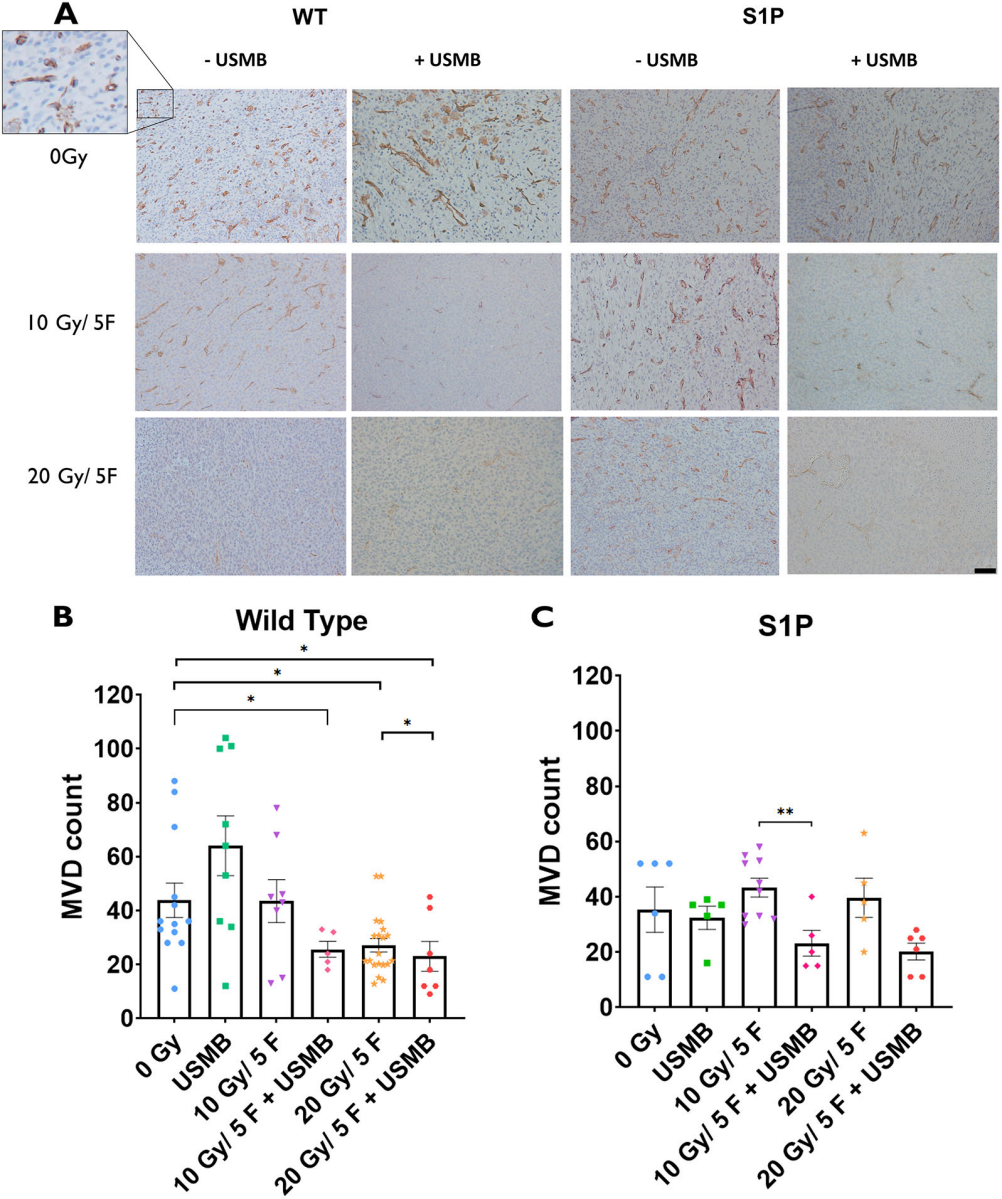
**Microvascular changes within MCA/129 fibrosarcoma tumours post radiation treatment in WT and S1P-treated mice.** (A) Anti-CD31 antibody staining of endothelial cells lining blood vessels allowed for visualization of MCA/129 mouse fibrosarcoma tumours in WT or S1P-treated mice. Scale bar: 40 µm. (B,C) Individual vessels were quantified to measure microvascular density (MVD) under a 10× objective lens for WT (B) and S1P-treated (C) groups. For WT mice: 0 Gy (*n*=13), 10 Gy/5F (*n*=8), 20 Gy/5F (*n*=20), USMB only (*n*=9), 10 Gy/5F+USMB (*n*=5), 20 Gy/5F+USMB (*n*=7). For S1P-treated mice: 0 Gy (*n*=6), 10 Gy/5F (*n*=10), 20 Gy/5F (*n*=5), USMB only (*n*=5), 10 Gy/5F+USMB (*n*=5), 20 Gy/5F+USMB (*n*=6). Error bars represent mean±s.e.m. Statistical analysis was using Welch's two-tailed unpaired *t*-test. *P<0.05; **P<0.005.

CD31 immunostaining of the tumor vasculature was used to evaluate microvascular density (MVD) by counting the number of stained vessels within five to ten fields of view under a 10× objective lens (number of vessels per 0.385 mm^2^) (see Materials and Methods). Histological analysis revealed a significant decrease in detected blood vessels in WT tumours receiving radiation of 20 Gy as fractionated doses (‘F’) over 5 days (4 Gy per day; denoted as 20 Gy/5F) (25.5±2.3 MVD) (*P*<0.05) compared to that of the untreated cohort (54.3±15.3 MVD) (Fig. 1A). However, tumours that were treated with 10 Gy/5F (43.5±8.0 MVD) appeared to retain similar MVDs that were comparable to those of the untreated cohorts.

Treatment with USMB alone resulted in an increase in MVD (61.0±12.4) compared to that of untreated cohorts (54.3±15.3). When combined with USMB, there was a visible decrease in the number of vessels in the 10 Gy/5F group (25.6±3.0 MVD) compared to that for fXRT alone (43.5±8.0 MVD) and a significant drop compared to that of the untreated cohort (*P*<0.05). This enhanced effect was not detected when the 20 Gy/5F treatment was coupled with USMB, but instead MVD levels were maintained (23.0±5.50), suggesting that 20 Gy/5F is capable of inflicting a maximal vascular damage effect that cannot be further enhanced (Fig. 1B).

Tumours treated with S1P appeared to convey the expected protective effect in non-USMB-treated cohorts, showing no significant difference between the 0 Gy (42.4±14.9 MVD), 10 Gy/5F (43.3±3.5 MVD) and 20 Gy/5F (39.6±7.2 MVD) groups. However, when combined with USMB, the respective fXRT treatments produced a decrease in MVD. USMB-fXRT treatment in the presence of S1P appeared to overcome the protective effect of S1P and induced a loss in MVD to a similar effect across both the 10 Gy/5F+USMB (23.2±4.7 MVD) and 20 Gy/5F+USMB (20.2±3.0 MVD, *P*<0.05) cohorts compared to the 0 Gy treatment (Fig. 1C). This enhancement in S1P cohorts does not appear to be dose dependent.

### Cellular apoptosis response to USMB and fXRT in WT and S1P-treated mice

**Fig. 2.**
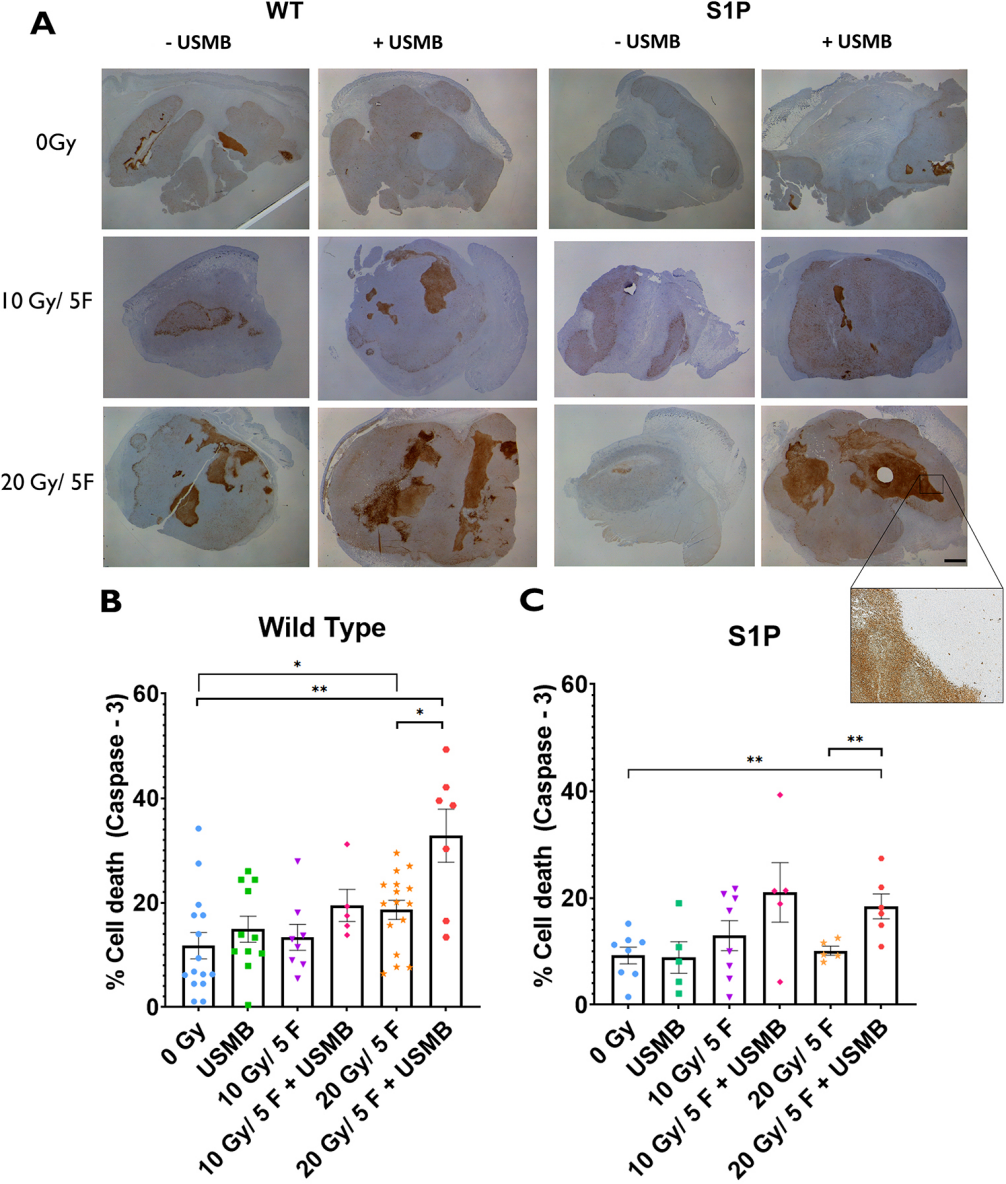
**Tumour cell death within MCA/129 fibrosarcoma tumours post radiation treatment in WT and S1P-treated mice.** (A) Immunohistochemistry staining for caspase-3 visualized whole areas of cell death within MCA/129 mouse fibrosarcoma tumours in WT or S1P treated mice. A dissection microscope at a 0.8× magnification was used to capture whole-tumour slices. Scale bar: 2 mm. (B,C) Whole-area staining was measured as total percentage of cell death detected in WT (B) and S1P-treated (C) groups. For WT mice: 0 Gy (*n*=15), 10 Gy/5F (*n*=8), 20 Gy/5F (*n*=16), USMB only (*n*=11), 10 Gy/5F+USMB (*n*=5), 20 Gy/5F+USMB (*n*=7). For S1P-treated mice: 0 Gy (*n*=8), 10 Gy/5F (*n*=8), 20 Gy/5F (*n*=5), USMB only (*n*=5), 10 Gy/5F+USMB (*n*=5), 20 Gy/5F+USMB (*n*=6). Error bars represent mean±s.e.m. Statistical analysis by Welch's two-tailed unpaired *t*-test. *P<0.05; **P<0.005.

Caspase-3 staining indicated a significant increase of cell death in response to the combination treatments (Fig. 2A). The 20 Gy/5F+USMB (32.8±5.1% of the tumour area stained for caspase-3) regimen showed significantly increased caspase-3 activity compared to that of the untreated (0 Gy, 14.1±3.3%, *P*<0.001) and 20 Gy/5F (18.6±2.0%, *P*<0.01) groups. The combination of 10 Gy/5F with USMB (21.0±5.6%) showed elevated levels of caspase-3 staining compared to that of 10 Gy/5F treatment alone (13.4±2.5%), with staining levels comparable to those of 20 Gy/5F alone (18.4±2.3%). The 0 Gy, USMB-only (14.4±3.2%) and 10 Gy/5F-alone cohorts showed similar levels of caspase-3 staining (Fig. 2B).

A similar result was observed in the S1P-treated cohorts in which fXRT-only cohorts (10 Gy/5F, 12.9±2.8%; 20 Gy/5F, 10.1±0.82%) showed no significant elevations of caspase-3 staining detected compared to that of untreated (9.72±1.71%) and USMB-only (8.86±2.95%) tumours, indicating a protective effect (Fig. 2C). However, the addition of USMB combined with 10 Gy/5F (21.0±5.6%) and 20 Gy/5F (18.4±2.3%) showed large areas of tumours with greater staining, indicating massive apoptotic events corresponding to the treatment. The effect does not appear to be dose dependent in S1P cohorts as the elevated levels of the combination treatments were comparable. The results observed in apoptotic cell death reflects the changes in MVD (Fig. 1).

### Cell proliferation assessment following USMB and fXRT in WT and S1P-treated mice

**Fig. 3.**
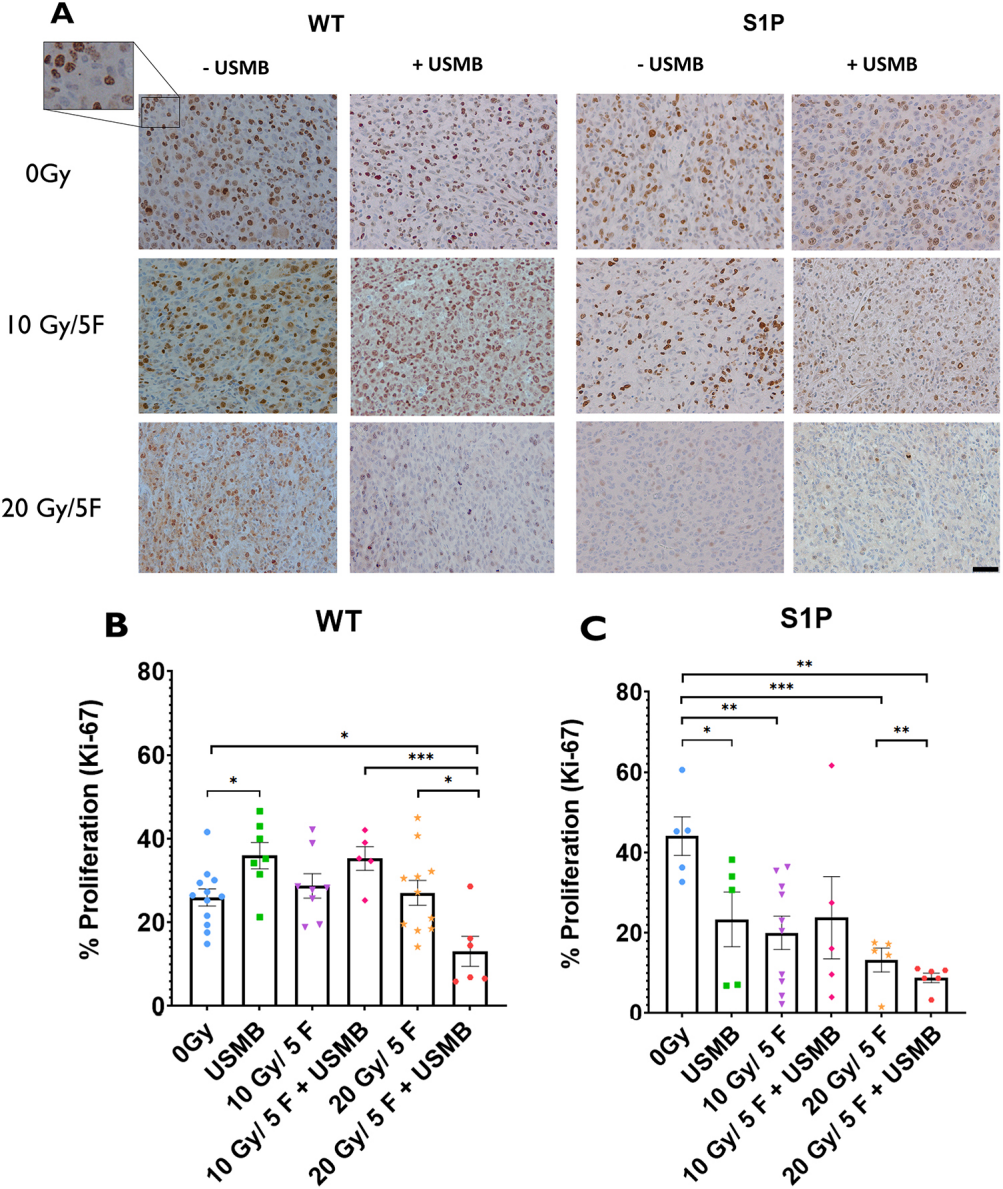
**Cell proliferation index within MCA/129 fibrosarcoma tumours post radiation treatment in WT and S1P-treated mice.** (A) Anti-Ki-67 antibody staining of proliferating primary tumour cells allowed for visualization of MCA/129 mouse fibrosarcoma tumours in WT or S1P-treated mice. Scale bar: 20 μm. (B,C) The numbers of individual cells of stained areas were calculated as a percentage of actively proliferating cells against the total cell count under a 20× objective lens of WT (B) and S1P-treated (C) groups. For WT mice: 0 Gy (*n*=12), 10 Gy/5F (*n*=8), 20 Gy/5F (*n*=11), USMB only (*n*=17), 10 Gy/5F+USMB (*n*=5), 20 Gy/5F+USMB (*n*=6). For S1P-treated mice: 0 Gy (*n*=5), 10 Gy/5F (*n*=10), 20 Gy/5F (*n*=5), USMB only (*n*=5), 10 Gy/5F+USMB (*n*=5), 20 Gy/5F+USMB (*n*=6). Error bars represent mean±s.e.m. Statistical analysis by Welch's two-tailed unpaired *t*-test. *P<0.05; **P<0.005; ***P<0.0005.

Ki-67 immunohistochemistry was used to ascertain the levels of active cell division within tumours (Fig. 3A). fXRT alone (10 Gy/5F, 28.7±2.9% of the tumour cells stained for Ki-67; 20 Gy/5F, 23.3±3.5%) did not have a significant effect on tumour proliferation rates in WT mice compared to those of untreated cohorts (0 Gy, 24.4±2.6%), whereas USMB alone (36.7±3.2%) resulted in a significant increase of proliferation activity. Treatment with 10 Gy/5F+USMB also showed an increase of proliferation activity similar to that of treatment with USMB alone; however, this change was not significant compared to untreated cohorts. Additionally, tumours undergoing 20 Gy/5F+USMB (18.1±5.9%) treatments showed a significant reduction in proliferation post treatment (*P*<0.01) (Fig. 3B).

The addition of S1P appeared to have a different effect on cell proliferation in an XRT-dose-dependent manner. Tumours that underwent fXRT, USMB treatment or a combination treatment showed significantly lower levels of proliferation across all cohorts compared to those of untreated controls (44.8±4.8%). Treatment with USMB only (23.4±6.8%), 10 Gy/5F (20.1±4.1%) and 10 Gy/5F+ USMB (23.8±10.4%) showed reduced proliferation rates at similar levels, whereas 20 Gy/5F treatment (13.3±3.0%) showed further reduced proliferation and 20 Gy/5F+USMB treatment (8.8±1.2%) showed the greatest reduction in proliferation, indicating that S1P, fXRT and USMB treatment have compounding effects on tumour proliferation in a dose-dependent manner (Fig. 3C).

### Effects of USMB and fXRT in ASMase^−/−^ mice

**Fig. 4.**
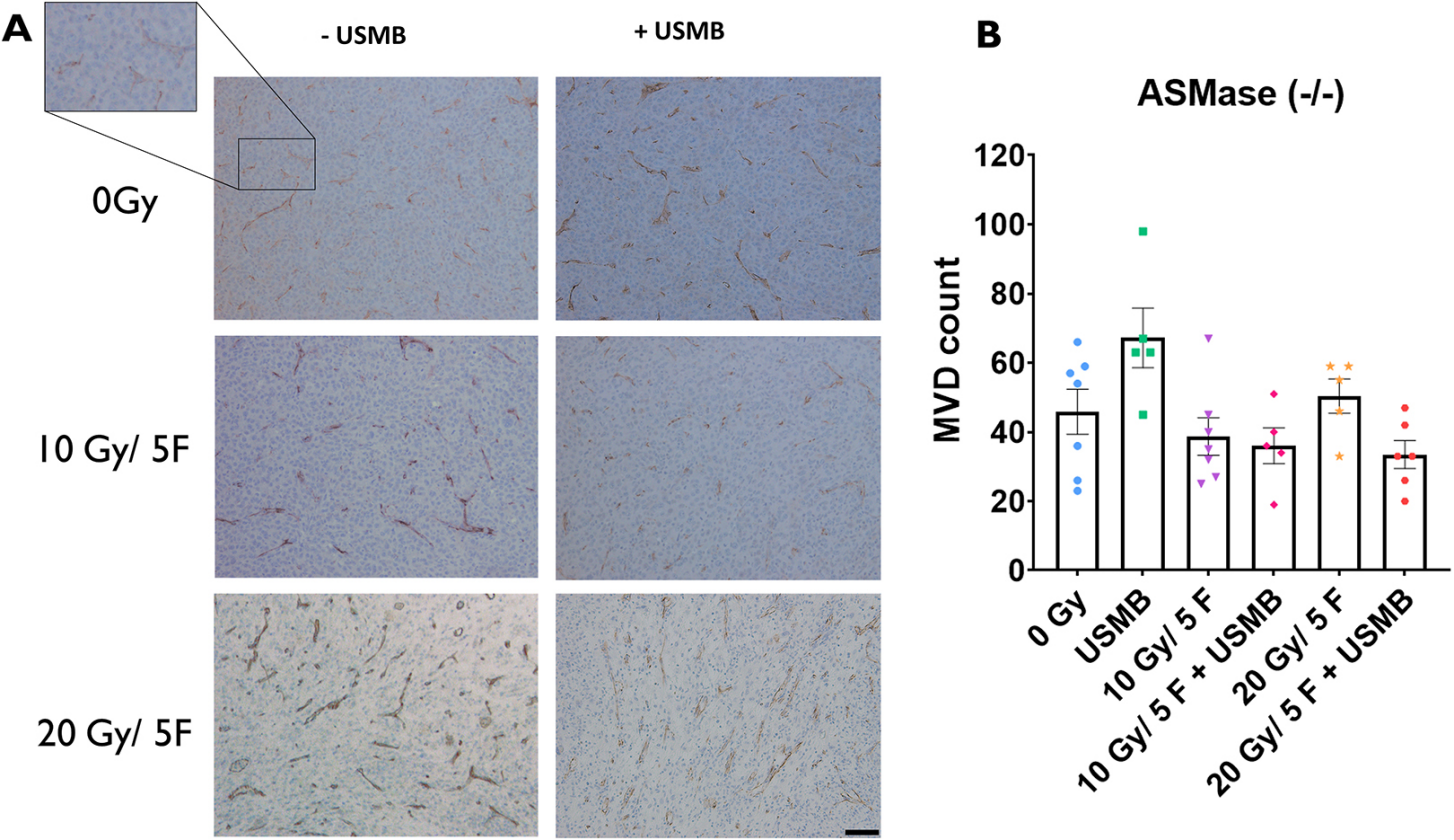
**Microvascular changes within MCA/129 fibrosarcoma tumours post radiation treatment in ASMase^−/−^ mice.** (A) Anti-CD31 antibody staining of endothelial cells lining blood vessels allowed for visualization of MCA/129 mouse fibrosarcoma tumours in WT or S1P-treated mice. Scale bar: 40 μm. (B) Individual vessels were quantified to measure MVD under a 10× objective lens. 0 Gy (*n*=7), 10 Gy/5F (*n*=7), 20 Gy/5F (*n*=5), USMB only (*n*=5), 10 Gy/5F+USMB (*n*=5), 20 Gy/5F+USMB (*n*=6). Error bars represent mean±s.e.m. Statistical analysis by Welch's two-tailed unpaired *t*-test.

Experiments conducted with ASMase^−/−^ cohorts showed no significant changes in response to treatments (Fig. 4A). The endothelial vasculature appeared to maintain constituency, showing no significant MVD decrease across all cohorts, even when tumours were treated with 20 Gy/5F+USMB. The USMB-only and 20 Gy/5F cohorts appeared to have increases in MVD (0 Gy, 45.9±6.5; 10 Gy/5F, 38.7±5.4; 20 Gy/5F, 50.4±5.0; USMB only, 67.2±8.6; 10 Gy/5F, 36.0±5.2; 20 Gy/5F, 33.5±4.1), but these were non-significant differences and might be a result of animal variability (Fig. 4B). These results reflect the findings from previous studies of ASMase^−/−^ tumours conveying radioresistance to endothelial cells (El-Kaffas et al., 2018).

### Cell death effects of USMB and fXRT in ASMase^−/−^ mice

**Fig. 5.**
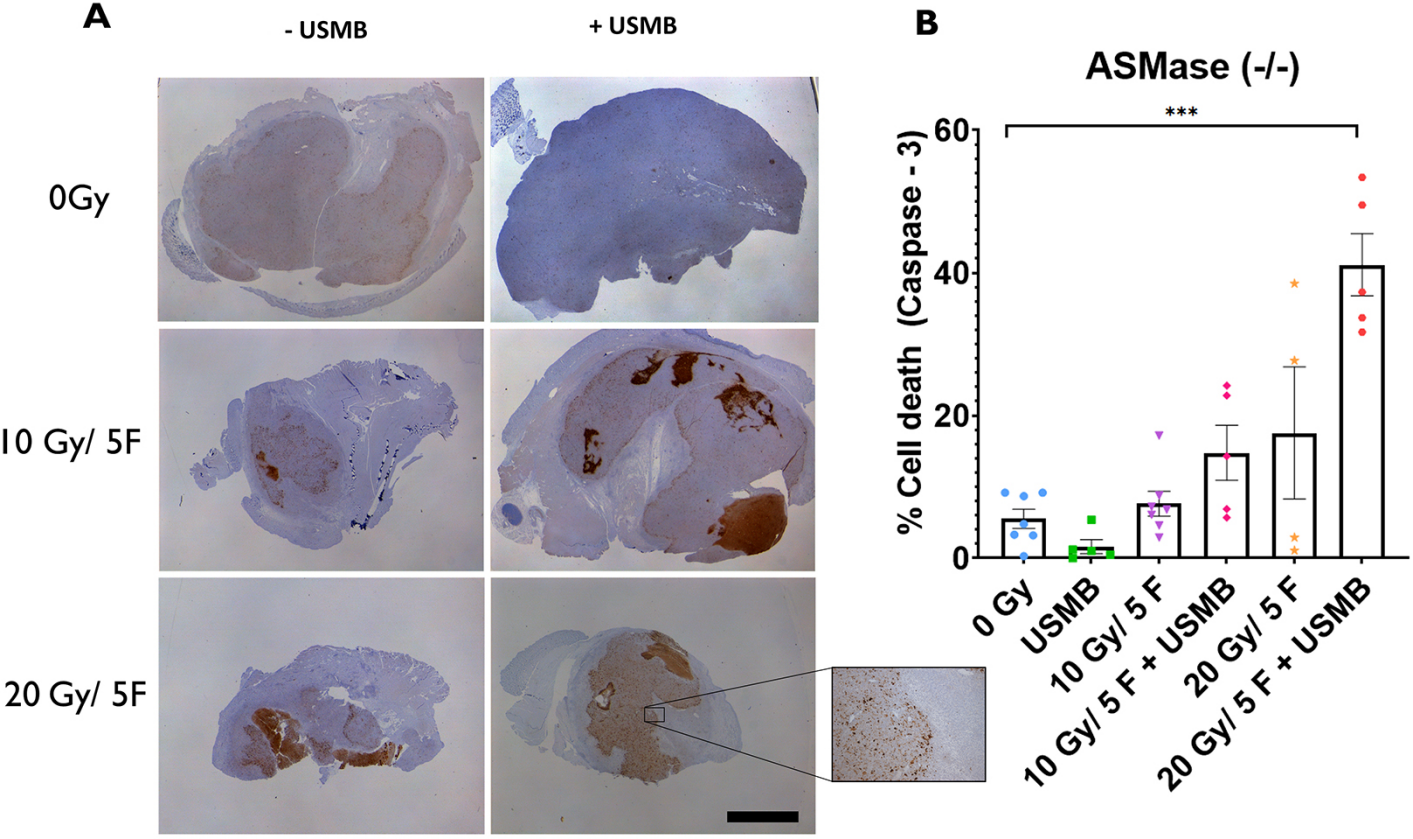
**Tumour cell death within MCA/129 fibrosarcoma tumours post radiation treatment in ASMase^−/−^ mice.** (A) Antibody staining for caspase-3 visualized whole areas of cell death within MCA/129 mouse fibrosarcoma tumours. Scale bar: 10 μm. (B) Whole-area staining was measured as total percentage of cell death detected. 0 Gy (*n*=8), 10 Gy/5F (*n*=8), 20 Gy/5F (*n*=5), USMB only (*n*=5), 10 Gy/5F+USMB (*n*=5), 20 Gy/5F+USMB (*n*=6). Error bars represent mean±s.e.m. Statistical analysis by Welch's two-tailed unpaired *t*-test. ***P<0.0005.

ASMase^−/−^ mice were anticipated to exhibit results similar to those of S1P-treated cohorts, in which primary tumour cells would exhibit a radioresistant phenotype and show reduced levels of cell death. However, in ASMase^−/−^ cohorts, caspase-3 activities were enhanced when tumours were treated with fXRT+ USMB compared to treatment with fXRT alone. Gross histology indicated an increasing level of response scaling to the intensity of treatment rather than a radioresistant phenotype (Fig. 5A). Treatment with USMB alone showed a decrease in caspase-3 activity compared to the 0 Gy (untreated) cohort, although this reduction was not significant. Tumours exposed to 10 Gy/5F (7.65±2.7% of the tumour areas stained for caspase-3) exhibited a twofold increase of caspase-3 activation when combined with USMB (14.8±3.9%), indicating an enhanced effect in which USMB causes caspase-3 activation of primary tumour cells. Additionally, these levels of enhanced caspase-3 activation following USMB treatment are comparable to those of caspase-3 activation with 20 Gy/5F alone (17.6±9.2%). We observed a similarly enhanced response in the 20 Gy/5F+USMB cohort (41.1±4.4%), suggesting that despite the preservation of the microvasculature as a result of ASMase knockout, primary tumour cell still responded to the fXRT+USMB treatments through a different mechanism (Fig. 5B).

### Cell proliferation in ASMase^−/−^ mice

**Fig. 6.**
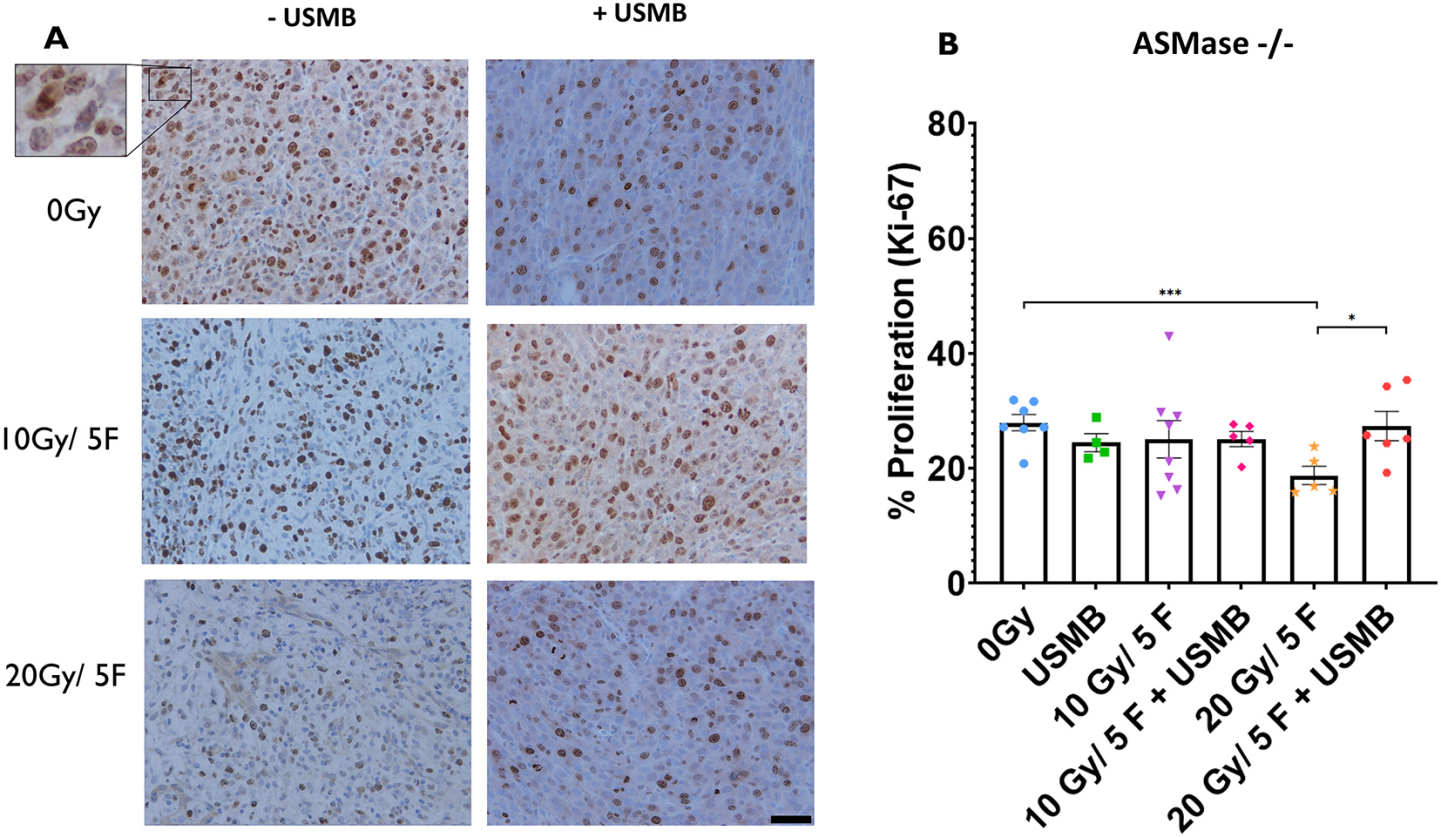
**Cell proliferation index within MCA/129 fibrosarcoma tumours post radiation treatment in ASMase^−/−^ mice.** (A) Anti-Ki-67 antibody staining of proliferating primary tumour cells. Scale bar: 20 µm. (B) Individual cell counting for stained cells as a percentage of actively proliferating cells against total cell count in one field of view under a 20× objective lens. 0 Gy (*n*=7), 10 Gy/5F (*n*=8), 20 Gy/5F (*n*=5), USMB only (*n*=4), 10 Gy/5F+USMB (*n*=8), 20 Gy/5F+USMB (*n*=5). Error bars represent mean±s.e.m. Statistical analysis by Welch's two-tailed unpaired *t*-test. *P<0.05; ***P<0.0005.

Rates of proliferation detected using Ki-67 immunohistochemistry demonstrated no significant changes across all treatment cohorts in ASMase^−/−^ mice (0 Gy, 28.0±1.4% of the tumour cells stained for Ki-67; 10 Gy/5F, 25.1±3.3%; 20 Gy/5F, 18.8±1.6%; USMB only, 22.6±2.2%; 10 Gy/5F+USMB, 25.2±1.3%; 20 Gy/5F+ USMB, 27.4±2.5%), with the exception of the 20 Gy/5F group showing a decrease in proliferation rates compared to those of the 0 Gy (*P*<0.002) and 20 Gy/5F+USMB (*P*<0.05) cohorts (Fig. 6A,B). Despite this significance, the similar grouping of values across all other conditions suggests that proliferation is not greatly affected by fXRT-alone, USMB-alone or fXRT combined with USMB treatments. This result more closely resembles the proliferation rates of the WT cohorts than those of the S1P cohorts.

### PD assessment in WT, S1P-treated and ASMase^−/−^ mice exposed to USMB and fXRT

**Fig. 7.**
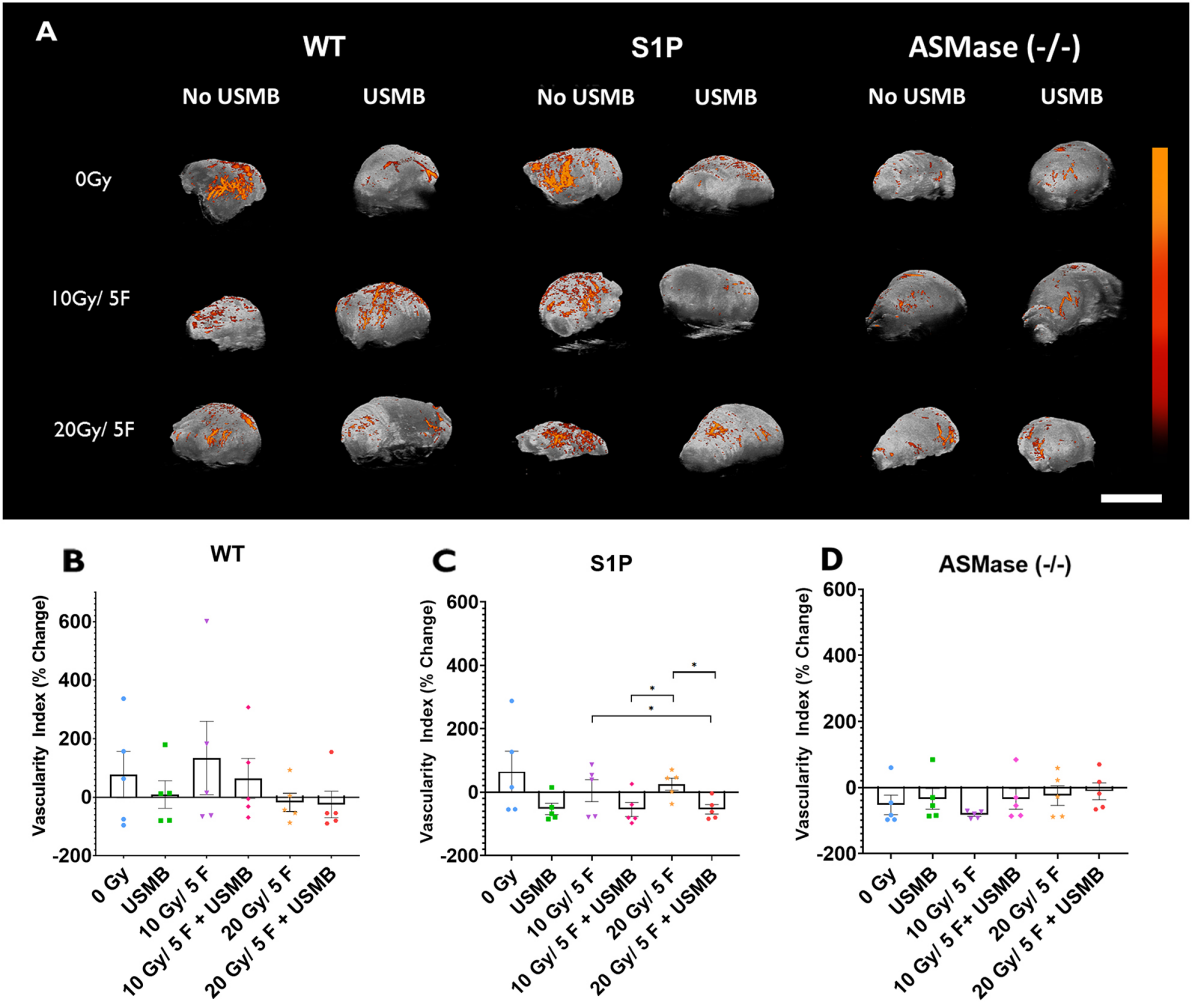
**Power Doppler analysis of tumours treated with fXRT and/or USMB.** (A) Maximum-intensity projections of power Doppler (PD) signals within a 3D volumetric tumour scan of whole tumours undergoing the indicated treatments. The colour bar represents a range from 11 dB (black) to 30 dB (orange). Scale bar: 1 cm. (B-D) Percentage change of the vascular index signal in each treatment condition for the WT, S1P-treated and ASMase^−/−^ groups. Each cohort had five mice. Results are represented as signal intensity change from baseline pre-treatment scan. Error bars represent mean±s.e.m. Statistical analysis by Welch's two-tailed unpaired *t*-test. *P<0.05.

The results collected with PD data revealed microvascular changes within tumours; the vascularity index (VI) measures the percentage change of signal detected pre- and post treatment of the tumours (Fig. 7A). In WT tumours, the 0 Gy (VI, 77.2±79.7), USMB-only (9.24±47.3), 10 Gy/5F (133.9±125.2) and 10 Gy/5F+USMB (63.9±68.5) groups showed increasing levels of blood flow after all treatment was complete; however, the 20 Gy/5F (−17.4±31.2) and 20 Gy/5F+USMB (−24.8±45.4) groups showed decreased blood flow as a result of the treatments. Despite this, statistical analysis revealed no significant changes across all treatment conditions (*P*>0.05) (Fig. 7B).

Groups treated with S1P showed a trend of decreasing blood flow upon treatment with USMB. The 0 Gy (64.3±64.9), 10 Gy/5F (4.83±34.5) and 20 Gy/5F (25.1±19.1) groups showed an increase of blood flow post treatment, whereas USMB-only (−52.9±18.2), 10 Gy/5F+USMB (–54.7±22.3) and 20 Gy/5F+USMB (–54.1±14.7) treatments showed decreases of vascular signal post treatment. However, only the 20 Gy/5F treatment regimen showed statistically significant difference compared to the USMB-only, 10 Gy/5F+USMB and 20 Gy/5F groups (Fig. 7C). Comparisons of each treatment in ASMase^−/−^ mice revealed overall decreases of PD signal post treatment under all conditions; however, none of these changes were statistically significantly different (Fig. 7D).

Overall, PD signals collected were of very low magnitudes; thus, percentage change calculations could potentially exaggerate large levels of change found within tumour volumes. Taken together with the results being non-significant after statistical analysis, the conclusions drawn from these data can only be speculative at best.

### Effects of USMB and fXRT on tumour growth delay in WT, S1P-treated and ASMase^−/−^ mice

**Fig. 8.**
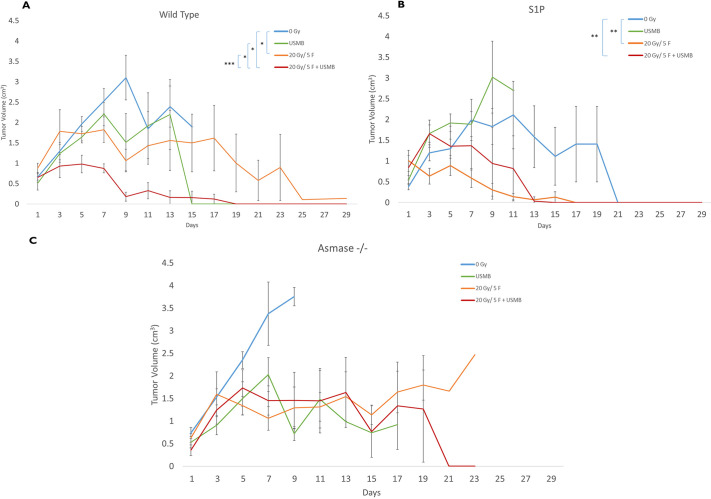
**Tumour growth curves over the course of 29 days.** Mice were treated on days 1-5 and were observed over the course of 29 days to observe tumour growth. (A) WT cohorts. (B) WT mice treated with S1P prior to fXRT or USMB treatments. (C) Mice with ASMase^−/−^ genotype. For WT mice: 0 Gy (*n*=11), 20 Gy/5F (*n*=5), USMB only (*n*=5), 20 Gy/5F+USMB (*n*=5). For S1P-treated mice: 0 Gy (*n*=9), 20 Gy/5F (*n*=5), USMB only (*n*=3), 20 Gy/5F+USMB (*n*=5). For ASMase^−/−^ mice: 0 Gy (*n*=5), 20 Gy/5F (*n*=5), USMB only (*n*=6), 20 Gy/5F+USMB (*n*=5). Error bars represent mean±s.e.m. Time points for which no error bars are present are points for which there was only one animal remaining for observation. Statistical analysis by Wilcoxon's two-tailed unpaired *t*-test. *P<0.05; **P<0.005; ***P<0.0005.

WT tumours treated with 20 Gy/5F or 20 Gy/5F+USMB showed growth suppression after treatment, with tumour volumes peaking at day 5 for the 20 Gy/5F cohorts (1.65±0.12 cm^3^) and day 3 for the 20 Gy/5F+USMB cohorts (0.90±0.24 cm^3^). Many of these mice continued to maintain or showed reduction in tumour size over the course of the subsequent days, with no rebound in tumour growth post treatment. Tumours that underwent 20 Gy/5F (*P*<0.05) and 20 Gy/5F+USMB (*P*<0.05) treatments showed significantly greater growth suppression compared to controls, whereas USMB treatment alone showed no significant differences. Furthermore, the combination treatments showed a significantly higher level of tumour growth suppression than that of fXRT alone (*P*<0.0001). Tumours that were not treated or treated with USMB only continued to rapidly increase in volume, tripling in size by the end of the second week, with peak tumour volume at day 9 for untreated cohorts (3.31±0.77 cm^3^) and at day 13 for USMB-only groups (3.39±0.74 cm^3^). It was also noted that most of the tumours in the 0 Gy or USMB cohorts reached the end-point size at an earlier time than tumours that were treated with fXRT or the combination treatment, and the mice were sacrificed before the end of the 29-day observation period (Fig. 8A).

In S1P cohorts, mice undergoing either 20 Gy/5F or 20 Gy/5F+USMB treatments showed similar trends of tumour growth decline post treatment, with peak tumour volume at day 1 for the 20 Gy/5F group (1.03±0.12 cm^3^) and day 5 for the 20 Gy/5F+USMB group (1.42±0.49 cm^3^) compared to controls (*P*<0.001). These contrasting results were seen in both the untreated and USMB-only cohorts, both of which showed continuous tumour growth with peak tumour volumes at day 19 for the 0 Gy group (2.51±0.75 cm^3^) and day 9 for the USMB-only group (2.96±0.50 cm^3^) (Fig. 8B).

Mice with the ASMase^−/−^ genotype showed similar tumour volumes across all treatment cohorts (USMB only, 20 Gy/5F and 20 Gy/5F+USMB), with tumour growth being halted and having relatively minor levels of regression over several days. Untreated cohorts (0 Gy), however, continued to grow at a rapid pace, nearly tripling in size by day 9 (3.98±0.30 cm^3^), resulting in early termination of all mice in accordance with research ethics guidelines (Fig. 8C). No significant differences were found among all conditions.

No mice under observation died as a result of treatment toxicity. Animals in which tumours grew beyond the end-point size (2.5 cm in length) or tumour burden began to cause distress and pain were sacrificed prematurely in accordance with ethical guidelines. Individual tumour growth curves can be seen in Fig. S1.

Overall, fXRT+USMB treatments appear to suppress tumour growth, resulting in stabilization post treatment with no apparent rebounding of tumour growth within 29 days.

## DISCUSSION

The work here explores for the first time the involvement of the ASMase-ceramide molecular pathway following the exposure of USMB and fXRT in genetically modified mice deficient for the ASMase-encoding gene *SMPD1*. The results of this study show that USMB treatment can be an effective radioenhancer in combination with fXRT and is capable of exhibiting greater tumour control compared to fXRT or USMB treatment alone. WT, S1P-treated and ASMase^−/−^ mice were treated with either 0 Gy, 10 Gy or 20 Gy in five fractions daily with or without USMB. USMB treatments were delivered twice a week on day 1 and 5 of the fractionation schedule with a 3% microbubble tail-vein injection stimulated by a 750 kPa pressure ultrasound field. Previous studies by Kolesnick and colleagues showed that exposing fibrosarcoma (MCA/129) and melanoma (B16F1) solid tumours to single doses of 20 Gy induced high levels of endothelial cell apoptosis in WT mice, whereas ASMase^−/−^ showed a radioresistant phenotype and reduced endothelial apoptosis (Garcia-Barros et al., 2003; Paris et al., 2001). In addition, the introduction of USMB to low single doses of XRT demonstrated the ability to enhance the cell-killing effect of 2 Gy and 8 Gy in prostate (PC3) xenografts in severe combined immunodeficiency disease (SCID) mice (Czarnota, 2015) and fibrosarcoma (MCA/129) murine allografts (El-Kaffas et al., 2018). Findings by El-Kaffas et al. (2018) revealed that USMB stimulation also enhanced endothelial apoptosis in WT mice but not in ASMase^−/−^ mice. These findings are reflected in our present results, in which WT mice showed high levels of cell death and MVD reduction 72 h post treatment, whereas the MVD in ASMase^−/−^ mice was relatively unaffected by the fXRT+USMB treatment. The results here showed an enhanced tumour response to fXRT when administered alongside USMB through the ASMase-ceramide pathway.

When 10 Gy/5F was combined with USMB, there was a loss of MVD equivalent to that the 20 Gy/5F treatment alone. This enhancement effect was similarly seen previously in the study by El-Kaffas et al. (2018), in which microbubble enhancement of 2 Gy XRT showed enhanced endothelial apoptosis comparable to that of the 8 Gy alone 72 h post treatment. The addition of high USMB concentration (3% microbubble solution) to the 8 Gy treatment, however, did not enhance the MVD loss compared to that of 2 Gy with high USMB treatment. This indicates that there is a maximal enhancement that USMB can apply alongside XRT that lies between 2 and 8 Gy. This effect is reflected in our findings as the 20 Gy/5F+USMB treatment showed a level of MVD reduction comparable to that of the 20 Gy/5F and 10 Gy/5F+USMB treatments. In a review by Fuks and Kolesnick (2005), individual dose delivery is important to elicit an ASMase-mediated apoptotic response that leads to tumour cell death. ASMase activation occurs only upon delivery of radiation >8 Gy. Indeed, previous studies have shown that single-dose exposures <8 Gy in CT26 colon cancer (Zhu et al., 2015) and MCA/129 fibrosarcoma (El-Kaffas et al., 2018) cells *in vivo* are not able to exhibit the cell-killing effect in an ASMase-dependent fashion. Given that USMB also enhances the apoptotic effect through ASMase activation and the generation of ceramide, it can be rationalized that at higher radiation doses, the enhancing effect of USMB is diminished when there is a saturation of ASMase activity or ceramide signalling. This idea is further enforced by the finding that the treatment of fXRT alone in mice treated with S1P failed to induce significant MVD reduction. However, when combined with USMB treatments, MVD was reduced to similar levels in both the 10 Gy/5F and 20 Gy/5F regimens. This shows that the manual activation of endogenous ASMase through USMB activity was sufficient to elicit MVD loss, but only when radiation was applied, as USMB treatments alone were not sufficient to cause MVD loss. When ASMase was knocked out completely, there was no MVD response even when treated with the combination treatment. This again highlights the importance of ASMase function in the induction of enhanced vascular disruption using USMB+fXRT, leading to tumour cell death.

Tumour cells in WT mice showed enhanced cell death when treatment intensities were increased, starting with the 10 Gy/5F+USMB treatment, with the greatest increase of caspase-3 expression in tumours treated with 20 Gy/5F+USMB. This increase of cell death corresponds with MVD loss in the 10 Gy/5F and the 10 Gy/5F+USMB treatments, in which the tumour response was enhanced as a result of USMB treatment. Lower doses of XRT (<10 Gy) are not high enough to induce a strong cell-killing effect as cell death signalling can be inhibited by the activation of the oxygen-sensitive hypoxia-inducible factor 1 (HIF-1) complex (Fuks and Kolesnick, 2005; Hamidi et al., 2022). HIF-1 mRNA transcripts are stored in cytosolic stress granules. When reactive oxygen species are generated as a result of hypoxia, the mRNA transcripts get transcribed to generate HIF-1. Activation of HIF-1 leads to the generation of vascular endothelial growth factor (VEGF), promoting angiogenesis to allow for reoxygenation and tumour growth. Thus, doses >8 Gy radiation are sufficiently high to induce endothelial apoptosis, which can overcome the survival signal generated by HIF-1 (Fuks and Kolesnick, 2005). This is seen in previous findings in which CT26 cells exhibited low cell death levels with a single dose of 6 Gy XRT. However, cell death levels increased when two or more doses of 6 Gy were administered 8 h post treatment, reaching a total dose >8 Gy (Zhu et al., 2015). In our study, we see a similar effect in the enhancement of tumour cell death and MVD loss with increasing fXRT dose. However, despite the fact that the levels of MVD loss were greatest when treated with 20 Gy/5F+USMB, the corresponding levels of cell death continued to be enhanced compared to the levels seen upon fXRT treatment alone. This suggests that, although there is a maximal effect that USMB+fXRT has on vascular endothelial cells, tumour cells are still affected by the combination treatment in a dose-dependent manner. This is the line of thought furthered by the results of the ASMase^−/−^ cohorts, as caspase-3 activation show greater increases in correspondence with increased treatment intensity, despite no reduction in MVD.

One possible explanation for this seemingly contradictory result is that the enhanced cell death effect can occur in an ASMase-independent fashion when USMB treatment is combined with fXRT. In previous studies by Moding et al. (2014), ATM deletion in the transgenic mouse model was capable of enhancing endothelial cell death when treated with single-dose (20 Gy) or fractionated (30 Gy/10F) XRT, resulting in enhanced cell death. Furthermore, treatments with curative doses of 50 Gy and 80 Gy/4F were capable of inducing growth delays and local control in primary sarcomas, but showed no enhanced cell death when comparing mice with a single ATM allele present to an ATM deletion (Moding et al. 2015). This was further examined by Torok et al. utilizing a similar transgenic mouse line to observe the effects of high single dose radiation treatments on primary murine lung cancer and findings also suggested that tumour cells, rather than endothelial cells, are the primary targets in facilitating tumour response in single-dose radiotherapy (Torok et al., 2019). Although we used lower doses of XRT in our study, the results seen in the ASMase^−/−^ cohorts suggest a similar phenomenon in which fXRT and/or USMB treatments enhance tumour cell death despite the apparent preservation of the tumour vasculature. As such, despite the basis of the study revolving around the theory that vascular disruption drives local tumour control, it cannot be ruled out that the enhancement effect could be a result of direct or indirect influences of fXRT+USMB to tumour cell interactions or, at the very least, acts independently of the ASMase-ceramide pathway in a fractionated radiation regimen. Further studies could be conducted to definitively confirm the driving mechanism for treatment enhancement.

Another potential explanation of the discrepancy between MVD preservation and increased cell death could be the vascular theory of radiation tumour cure. Given that the complete knockout of ASMase prevents endothelial cells from undergoing apoptosis even after taking damage from the treatments, dysfunctional endothelial cells would still remain but be unable to carry out normal endothelial function. This could lead to a reduction in perfusion within the tumour as the vascular architecture becomes compromised. Perfusion decreases as a result of USMB microvascular damage, and this has been noted to cause tumour cell death, despite the presence of endothelial cells (El-Kaffas et al., 2018; Li et al., 2020). One important point that should be noted is that CD31 staining of endothelial vascular cells only indicates the presence or absence of these cells to calculate MVD but does not account for the function and viability of these cells. This also does not indicate if the microvessels are perfused or non-perfused. Thus, the potential for damaged endothelial cells to exhibit pro-apoptotic signals could be a contributing factor to the enhanced tumour cell death seen in fXRT+USMB cohorts in addition to poor perfusion.

Additionally, PD analysis demonstrated that perfusion changes in tumours can be detected when WT tumours are treated with radiation and additional treatment with USMB can cause reduced perfusion levels within tumours. Tumours treated with 10 Gy/5F showed a lower level of blood flow when treated with USMB compared to fXRT alone, indicating that there is an effect in reducing perfusion within tumours. This corresponds with the histology findings in which vasculature loss contributes to reduced perfusion. Similarly, tumours treated with 20 Gy/5F showed a decrease in perfusion compared to that in tumours treated with 10 Gy/5F+USMB, but the addition of USMB did not enhance the perfusion loss. This is reflective in the state of the vasculature as seen in the histology. In S1P-treated mice, we observed a similar effect in that perfusion levels remained similar among the 0 Gy, 10 Gy/5F and 20 Gy/5F treatments, but the combination of USMB showed a drop of perfusion, reflective of histological findings. However, results obtained from ASMase^−/−^ animals demonstrated very little perfusion within tumours to begin with and, as such, it was difficult to detect major changes within the tumour between treatments. Histological findings revealed that the majority of the preserved vasculature within the tumours had vessel diameters ranging from 5 to 15 μm, suggesting that ASMase^−/−^ tumour vascular architecture consists primarily of capillary networks. This is in contrast to findings by El Kaffas et al. (2005) in which perfusion was well characterized through PD analysis. The Vevo 770 ultrasound system is capable of capturing signals within a vessel of 50 μm or greater diameter with a flow rate of >1 mm/s. Given that the majority of capillary networks have an average diameter of 5-10 μm, perfusion changes in the microvasculature can be missed. The use of newer and more sensitive machines, such as the Vevo 3100, can be used to overcome this hurdle and provide a greater characterization of perfusion within tumours. Additionally, the use of contrast enhancement agents could also be used to enhance PD signals to observe vascularity and perfusion changes. Work done by Eisenbrey and Forsberg (2010) showed that microbubbles are an effective contrast agent in monitoring angiogenesis by utilizing maximum-intensity projections to capture the complex architecture in murine xenografts, with different variations of this contrast agent capable of targeting angiogenic markers. Despite the hardware limitation, the characterization of perfusion within tumours in the WT and S1P cohorts still provide us with complimentary results to the established CD31 histology, and provide an additional modality to observe the effects that USMB+fXRT have on the vasculature and perfusion.

Despite the promising results that support the hypothesis that USMB can be used alongside fXRT to enhance tumour control, this model system has several limitations. It does not take into account the impact that the immune system has on the efficacy of this combination treatment. Cyclosporine A is a well-established immunosuppressant and has been characterized in exhibiting toxicity (Patocka et al., 2021). Although the doses of cyclosporine A administered in this study were well below lethal dosages, the effect of immunosuppression of mice cannot be ignored as many reports indicate that immune cells and the immune responses plays an important role in facilitating radiation-induced anti-tumourigenic effects (Derer et al., 2015; Carvalho and Villar, 2018; Liddicoat and Lavelle, 2019). However, despite the reduced immune response as a result of daily cyclosporine A treatments, fXRT and combination treatments were still capable of inducing a cell death response in both tumour and endothelial cells. Reduced tumour growth with 20 Gy/5F and 20 Gy/5F+USMB combination treatments were observed, suggesting that tumour damage still occurred despite an artificially suppressed immune system. Whether this is an independent mechanism of tumour cure or the ability of the remaining active immune system to still function despite its suppression is not clear. Future studies should be conducted to look into the changes in the immune response when tumours are treated with radiation alone or in combination with USMB to determine the impact the immune system has in positively or negatively influencing this treatment.

In summary, the findings presented here show the importance of the ASMase-ceramide pathway in facilitating the combined effects of low-dose fXRT and USMB on the tumour microenvironment. Having previously identified that XRT+USMB treatments in single doses are capable of enhancing tumour cell-killing effects up to 72 h after treatment (Czarnota, 2015; El Kaffas et al., 2005), the results here show that this effect can be replicated with fXRT, with similar effects seen up to 72 h after total treatment. Additionally, the use of PD as a non-invasive modality to capture tumour perfusion can be an excellent tool to compliment histological analysis and potentially monitor tumour progression through treatment. However, the enhanced cell death effect observed in ASMase^−/−^ cohorts, despite the preservation of the tumour vasculature, suggests that radiation-induced tumour cell death in fractionated regimens could be driven in an ASMase-independent fashion.

## MATERIALS AND METHODS

### Cell and tumour growth

MCA/129 mouse fibrosarcoma cell lines [obtained from Dr Zvi Fuks' and Dr Richard Kolesnick's laboratories (Memorial Sloan-Kettering Institute for Cancer Research)] were used for this experiment in accordance with the Sunnybrook Research Institute's research ethics board’s approval. Cells were grown in Dulbecco's modified Eagle medium (DMEM) with 4.5 g l^−1^ glucose, L-glutamine and sodium pyruvate (Wisent, Quebec, Canada; 319-005-CL) and mixed with 1% penicillin/streptomycin (Gibco, Grand Island, NY, USA; 15140-122) and 10% (v/v) characterized fetal bovine serum (HyClone, Logan, UT, USA; SH30396.03). Cells were incubated in an incubator at 37°C with 5% CO_2_ levels maintained in culture and monitored for any forms of contamination. The medium was changed every 3-4 days or when the medium decreased in pH, as indicated by colour change of the medium. Cells were split when fully confluent often at approximately 7 days post seeding using 3 ml 0.05% trypsin-EDTA (Gibco, 25300-054) in a large culture flask. No cell cultures used for tumour inoculation were greater than passage 8. Cell counting using a haemocytometer was conducted prior to injection to ensure an accurate concentration of 3 million cells per 100 µl injection. Cell pellets were suspended in a 1 ml solution of PBS+ (PBS with calcium and magnesium; Wisent, 311-011-EL) and further diluted in a 1:10 ratio of PBS+. Then, 10 µl of the diluted cell solution was placed on a haemocytometer and counted. Cells were proportionally calculated as per the cell counting protocol. The total cell count in a 1 ml volume was calculated as: number of cells×100×10,000, where multiplication by 100 accounts for dilution and 10,000 accounts for haemocytometer calibration.

An in-house-bred ASMase-deficient C57BL/6 (black 6) mouse line was used as our *in vivo* model producing three distinct genotypes: WT (+/+), heterozygous (+/−) and homozygous knockout (−/−) (Hua and Kolesnick, 2013). Both male and female mice were selected between the age range of 8 and 12 months for WT mice and 6 and 8 months for ASMase^−/−^ mice. These mice were supplemented with a 1:10 dilution of cyclosporine-A (15 mg kg^−1^ per day; Novartis, 00078010901) in 0.1% saline, starting at least 48 h before the first day of cell injection to ensure sufficient immunosuppression. Cyclosporine A (150 µl) was injected subcutaneously dorsally to the mouse on a daily schedule through treatment until the day of sacrifice. Selected WT and ASMase^−/−^ mice were used as tumour host mice for experimentation, whereas heterozygous cohorts were used as breeder mice to further generate ASMase^−/−^ mice. All experiments complied with Sunnybrook Research Institute's (SRI) ethical guidelines involving animal welfare and were approved by the SRI Research Ethics Board.

Approximately three million MCA/129 cells suspended in PBS were injected into the right hind leg of mice of ages ranging between 8 and 12 weeks old. Tumours were left to grow for 1 week, until they reached a diameter of 8-10 mm in length, before beginning imagining and treatment regimens.

In addition to the WT and the knockout cohorts, an additional cohort was added consisting of WT mice pre-treated with sphingosine-1-phosphate (S1P) (Enzo Life Science, Farmingdale, NY, USA) to inhibit ceramide activity. WT mice were injected with 100 mg µl^−1^ S1P in a PET solution (5% polyethylene glycol, 2.5% ethanol and 0.8% Tween 80) 30 min before treatment began to ensure sufficient absorption of the endogenous chemical.

### Treatments

The treatment timeline (Fig. S2) lasted 5 days regardless of the treatment regimen. Microbubble treatments were administered on day 1 and day 5 before radiation treatment, in which mice were irradiated immediately after exposure to microbubbles. Mice were anaesthetized throughout treatment and imaging using isofluorane (Fresenius Kabi, Toronto, Canada; CP0406V2), which was administered using a calibrated anaesthesia machine (Benson Medical Industries, Toronto, Canada; 07-8914722). On day 1, B-mode ultrasound and PD data were acquired before treatment began. Radiation treatments (XRT) were conducted every day for 5 days as fractionated doses. Mice were divided into one of three groups with different radiation regimens: 0 Gy, 10 Gy/5F or 20 Gy/5F. Additionally, each of these conditions was further subdivided into additional treatments with a 150 µl solution of 3% (v/v) Definity microbubbles (Lantheus Medical Imaging, Montreal, Canada; 515311-0608) in 100 μl of saline (Hernot and Klibanov, 2008) followed by a 150 µl saline flush. USMBs were activated using the VIALMIX (Lantheus Medical Imaging) activation device for 45 s to generate microbubbles. Microbubbles and flush were administered via tail vein catheter injection. Microbubble stimulation consisted of exposure to an ultrasound field generated by a 500 kHz single element transducer with a peak negative pressure of 750 kPa (Kim et al., 2013). Water was heated to and maintained at a temperature of 37°C before positional calibration of the natural peak focus of the transducer and treatment began. Tumours were centred at the focal distance of the transducer, with a focus area of approximately ∼1 cm diameter. Calibration was performed with a vertical and horizontal needle to ensure tumour positioning was accurate. A schematic diagram of USMB treatment setup is provided in Fig. S3.

Tumour volumes were measured pre-treatment and post treatment manually using callipers capturing the estimated length, width and height. Changes in tumour volumes were calculated with the following formula:

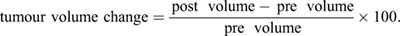
Mice were then given a 72 h recovery period after the final treatment before the sacrifice and the tumours were excised for histological analysis. Additionally, to examine the effect of combination treatments on tumour size and growth delay, an additional group of mice was used (*n*=60).

Four treatment regimens (0 Gy, USMB only, 20 Gy/5F or USMB+20 Gy/5F) were used to treat mice with a WT or ASMase^−/−^ genotype, or WT mice treated with S1P. Tumours were measured pre-treatment on day 1 and were subsequently measured every two days for a total of 29 days or until tumour diameter reached a dimension of >2.0 cm, while being administered cyclosporin A daily. Tumour volume was calculated by the following formula:

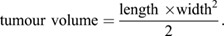


Mice were sacrificed early in case tumour sizes grew beyond the endpoints or showed signs of near mortality or suffering and it no longer acceptable to continue with the study in accordance to the research ethics protocol.

### Radiation treatments

Immediately after ultrasound exposure, mice were treated with radiation at a rate of 200 cGy min^−1^. Radiation regimens were conducted every day for 5 days as fractionated doses with a Faxitron cabinet irradiator (Faxitron Bioptics, Lincolnshire, IL, USA). X-rays at an energy of 160 kVp with a source-to-skin distance of 35 cm were administered to the target tumour volumes. A full-body lead shield was used to protect the mice with a 2.5 cm diameter hole to expose tumours to ionizing radiation. Mice were divided into one of three groups with different radiation regimens: 0 Gy (control), 10 Gy/5F or 20 Gy/5F, with individual dose administrations of 0 Gy, 2 Gy and 4 Gy, respectively.

### Ultrasound imaging

PD data were collected on days 1 and 8, before and after treatment, respectively. The right hind legs of mice, where the tumours were inoculated, were treated with a depilatory agent to remove fur covering the tumour region. Mice were then anesthetized using 5% isoflurane delivered via a head-covering nose-cone and were placed on a warming pad to maintain body temperature. The right leg was then fully submerged in a water bath and cleaned of bubbles or debris for scanning. A Vevo 770 ultrasound machine (Visualsonics, Toronto, Canada), using a 710B probe (Visualsonics) with a 30 MHz central frequency (15-45 MHz imaging band), 12.7 mm focal length and 55 µm resolution, was used to acquire 3D ultrasound and PD data. Once data were acquired, mice were returned to their cages, which were warmed by a heat lamp.

### Histology and immunohistochemistry

Histological analysis involved staining excised tissue samples with one of several tissue stains to observe morphological changes and/or differences between tumour samples and treatment conditions. Immediately after sacrifice, excised samples were divided into two portions for preservation. One half was fixed in 10% formalin (Thermo Fisher Scientific, SF93-4) for a minimum of 48 h, before being submerged in 70% ethanol until paraffinization of the sample was possible. The other half was submerged in Tissue-Tek Optimal Cutting Temperature compound (Sakura Finetek USA, Torrance, CA, USA) before being wrapped in foil and submerged in liquid nitrogen to flash freeze tissue samples. Frozen samples were stored at −80°C until further use was required.

Formalin-fixed paraffin-embedded tumour samples were sectioned and stained by the Pathology Research Program (PRP) Laboratory at the University Health Network (Toronto, Ontario, Canada). All antibodies and reagents for the respective stainings were provided by the PRP Laboratory. Tumour samples were cut into 5 μm-thin slices used for different visualization of tissue structures and protein activity. Haematoxylin and Eosin staining of tissue samples was used to observe structural and morphological changes within the tumour structure. The anti-Ki-67 antibody (1:1500, Abcam, ab15580) was used to stain cells undergoing proliferation, and was detected using the MACH 4 kit (Biocare, BC-M4U534L). Anti-CD31 immunohistochemistry (1:200, Novus Biologicals, NB-100-2284) was used to observe vascular endothelial cells and tumours vasculature. The anti-caspase-3 antibody (1:600, Cell Signaling Technology, 9661) was used to observe areas of tumours undergoing early stages of apoptosis. The anti-CD31 and anti-caspase-3 antibodies were detected using the ImmPRESS anti-rabbit kit (Vector Laboratories, MP-7401). Samples were then observed under a light microscope at 10× objective magnification for CD31 and 20× for Ki-67 visualization, and under a dissection microscope at 0.8× magnification for observing whole-tumour caspase-3-stained areas.

Quantification of Ki-67 and CD31 was conducted using ImageJ (v 1.53e) (https://imagej.nih.gov/ij/) for cell counting. Ten images were captured for Ki-67 and CD31 staining at 10× and 20× magnification, respectively, across tumour samples evenly to avoid area-capture bias. For the quantification of the percentage of cell proliferation, each image was processed using ImageJ to count proliferating cells, which were stained brown with Ki-67 immunohistochemical staining, and identify total cell number within the tumours. RGB images were split into separate black-and-white channels to generate contrast between the blue staining of background cells and brown stains of stained cells. The blue channel was used for filtering contrast of converted dark pixels for processing, whereas the red channel was used for filtering contrast of converted dark pixels of background cells. After manual curation to separate individual cells, cells were automatically counted using the built-in ‘Analyze Particle’ function in ImageJ. Once the total number of stained cells and total cell number was obtained, the proliferation index was calculated with the following formula:

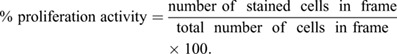


The average percentage of proliferation represented per frame was calculated in order to produce a representative percentage proliferation for each sample. Once all frames for a tumour sample were calculated, the total average of frames was summed and averaged. Per cohort, the total average sample was calculated for final evaluation.

The plugin ‘Cell Counter’ was added to ImageJ to permit a more accurate manual counting of blood vessels observed through staining of CD31. MVD was measured by counting the number of CD31-stained vessels observed within a given field of view (vessels per 0.385 mm^2^) under a 10× objective lens. For each tumour sample, five to ten fields of view were acquired and the mean vessel count was used to represent the tumour sample. A single vessel was characterized by intense staining showing a vascular track including connection branches or circular stains, indicative of a cross-sectional cut.

Caspase-3 staining analysis was conducted using a low-magnification dissecting microscope to capture whole-tumour stains. Tumour regions were manually selected in an in-house-written staining analysis program (MATLAB vR2011a), in which areas of brown stains indicative of cell death were detected and the percentage of cell death was calculated.

### Statistical analysis

Statistical analysis used GraphPad 8 (v9.0.0.121) to observe significance between treatment conditions. Multiple two-tailed unpaired Welch's *t*-tests were conducted between different treatment groups using a *P*<0.05 to indicate statistical significance. For tumour growth curves, multiple two-tailed unpaired Wilcoxon *t*-tests were conducted to compare significant changes between treatment groups paired with corresponding days.

## Supplementary Material

10.1242/dmm.049531_sup1 Supplementary information
