# Supplementary material for: Ultrasound-stimulated microbubbles enhanced vascular disruption in fractionated radiotherapy-treated tumours via ASMase activation
Source: Dis Model Mech. 2023 Jun 6;16(6):dmm049531. doi: 10.1242/dmm.049531 (PMC10259839; doi:10.1242/dmm.049531)
Supplement: Supplementary information [file dmm-16-049531-s1.pdf]

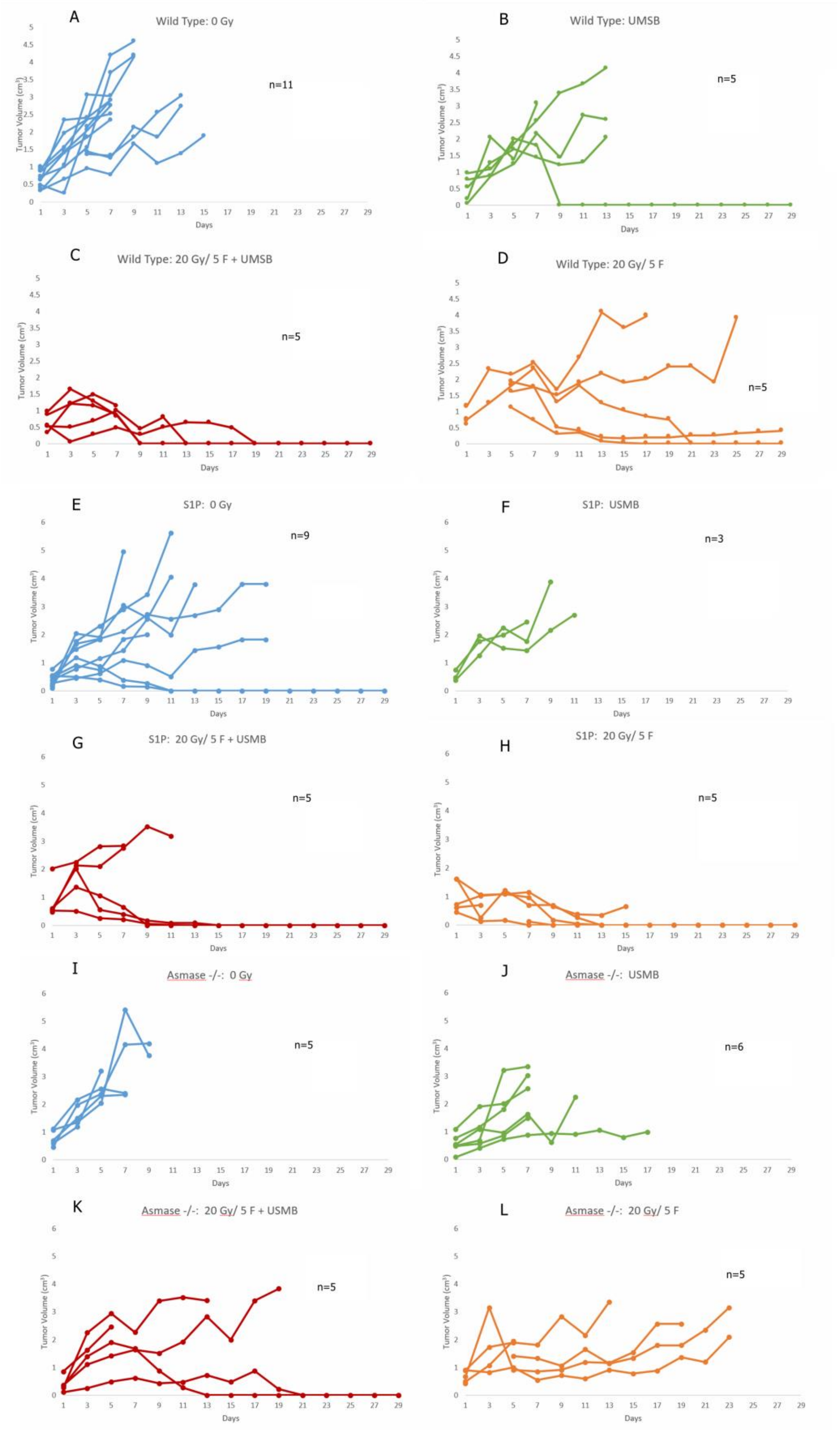

Disease Models & Mechanisms • Supplementary information

Fig. S1. Tumour growth curves of individual tumours over the course of 29 days. Individual tumour growth curves for each genotype and treatment. Mice were treated with USMB only, 20 Gy/ 5 F only or 20 Gy/ 5 F + USMB. fXRT treatment was done on days 1-5 and were observed over the course of 29 days to observe tumour growth. (A-D) Wild-type cohorts. (E-H) Wild-type mice treated with S1P prior to fXRT or USMB treatments. (I-L) Mice with ASMase -/- genotype. WT: 0 Gy (n=11), 20 Gy/ 5F (n=5), USMB only (n=5), 20 Gy/ 5F + USMB (n=5). S1P: 0 Gy (n=9), 20 Gy/ 5F (n=5), USMB only (n=3), 20 Gy/ 5F + USMB (n=5). ASMase -/-: 0 Gy (n=5), 20 Gy/ 5F (n=5), USMB only (n=6), 20 Gy/ 5F + USMB (n=5). Mice which did not make it to the end of 29 days were sacrificed early due to tumour burden reaching endpoint (2.5 cm diameter). Days which there are no data points are days which data was missing.

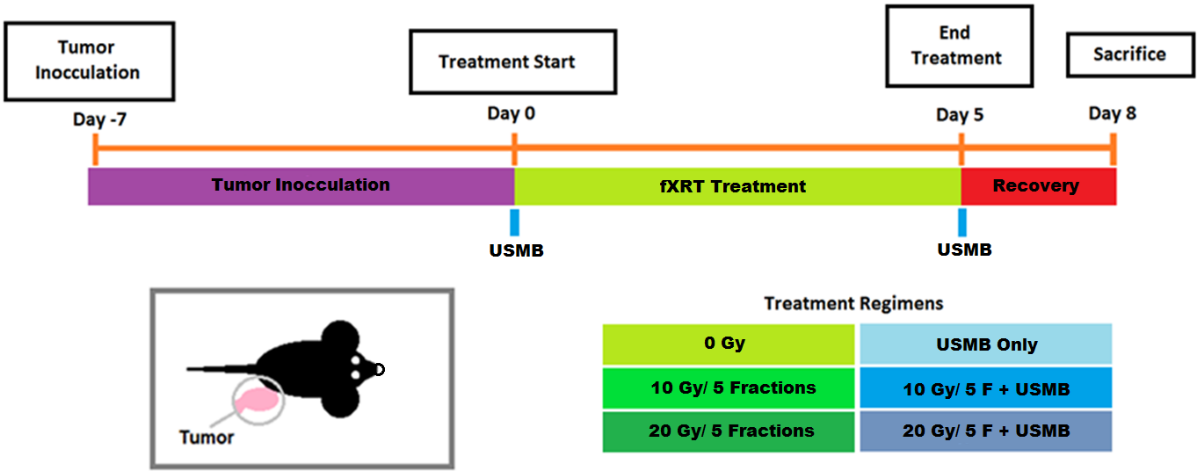

Fig. S2. Treatment regiment of fXRT. Mice were inoculated in the right hind leg and left for 7 days to grow to a size of 0.8-1 cm diameter before treatment begins. Mice were divided up into one of six groups; 0 Gy, USMB only, 10 Gy/ 5 F, 10 Gy/ 5 F + USMB, 20 Gy/ 5 F or 20 Gy/ 5 F + USMB. XRT was administered every day for 5 days at 2 Gy/ day or 4 Gy/ day while USMB treatments were delivered on day 1 and day 5. Mice were left to recover for 72 hours before sacrifice.

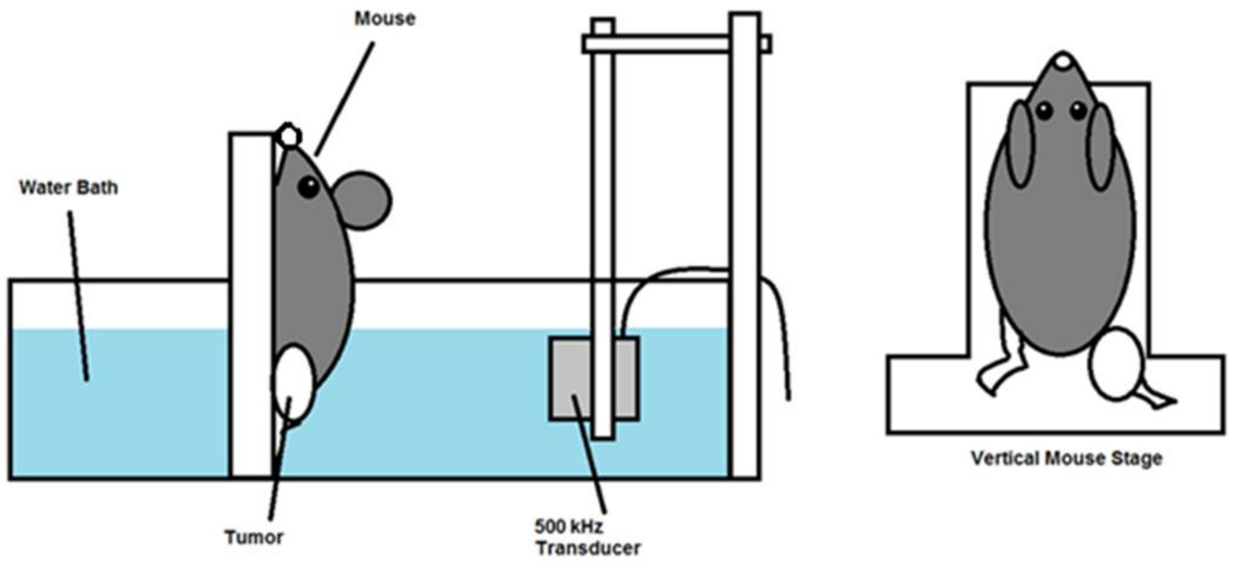

Fig. S3. Microbubble Setup. Tumours was fully submerged in the water bath heated to 37℃. A 500 kHz single element transducer was used to activate microbubbles and tumours was placed at transducer's focus. Tumours were exposed to ultrasound fields for 5 minutes immediately after injection of 3% USMB via tail vein catheter.

Disease Models & Mechanisms • Supplementary information
